# ﻿Morphological characteristics and phylogenetic analyses revealed two new species from China and a new record from Jilin Province of Agaricales

**DOI:** 10.3897/mycokeys.109.128960

**Published:** 2024-09-26

**Authors:** Di Zhang, Jun-Jie Peng, Jia-Jun Wang, A. K. Hasith Priyashnatha, Jin-Peng Liao, Hua-Xing Luo, Shi-Chen Huang, Ji-Ze Xu

**Affiliations:** 1 Agricultural College, Yanbian University, Yanbian 136200, China JiLin Agricultural Science and Technology University Jilin China; 2 Agricultural College, JiLin Agricultural Science and Technology University, Jilin 132000, China Yanbian University Yanbian China; 3 Department of Biology, Faculty of Science, Chiang Mai University, Chiang Mai 50200, Thailand Chiang Mai University Chiang Mai Thailand; 4 Management Bureau of Tianbaoyan National Nature Reserve in Yong'an, Yongan 366000, Chian Management Bureau of Tianbaoyan National Nature Reserve in Yong'an Yongan China; 5 Yong'an Zhisheng Chemical Union Co., Ltd, Yongan 366000, China Yong'an Zhisheng Chemical Union Co., Ltd Yongan China

**Keywords:** *Clitolyophyllum*, morphological characteristics, new species, phylogenetic analyses, *Stropharia*

## Abstract

In this study, we have found two new species—*Strophariasubrugosoannulata* and *Strophariamicroaeruginosa*. Phylogenetic analyses, based on the internal transcribed spacer regions (ITS) and the nuclear ribosomal RNA gene (nrLSU), suggest that the two new species are distinct and monophyletic. *S.subrugosoannulata* is distinguished from other species of the genus *Stropharia* by the pileus covered with greyish-orange squamules in the centre, stipe light brown and surface covered with white triangular squamules. *S.microaeruginosa* differs from other species in its pileus bluish-grey when young becoming lighter towards margins, later greyish-turquoise lightens towards the edges and surface radially striate when young, lamellae adnate to subdecurrent, stipe with white squamules at the base, acanthocytes absent. The new record species from Jilin Province, *Clitolyophyllumumbilicatum* was also confirmed, based on morphological and molecular study. Here, we have given full descriptions of each species, colour images, illustrations and two phylogenetic trees that show the placement and relationship of the two new species and the new record are provided.

## ﻿Introduction

*Stropharia* (Fr.) Quél. is the type genus of the family Strophariaceae Singer & A.H. Sm. Species of *Stropharia* are characterised mainly by pileus with viscid to dry, slightly hygrophanous or not, glabrous to floccose to squamous surface, a central, cylindrical, viscid or dry stipe often with a distinct annulus or annular zone, lamellae are dark purple, purplish-grey, or brownish-grey and the base of the stipe has white rhizoids (Murrill 1918, 1922; Hawksworth et al. 1983). Many species in this genus are well-known medicinal fungi, such as *Strophariarugosoannulata* Farl. ex Murrill *Strophariacubensis* Earle and others (Dai et al. 2012).

Fries (1821) had recognised three clades within *Agaricus* and placed *Stropharia* in Tribus *Psalliota*. In order to accommodate a number of morphologically distinct species within *Agaricus*, Fries (1849) established subgenus Stropharia. Singer and Smith (1946) were raised to the genus level of subgenus Stropharia in 1946. The 7^th^ and 8^th^ editions of the Dictionary of the Fungi also adopts the classification of Singer et al. (Hawksworth et al. 1983, 1995). However, Smith (1979) changed his view and later on, together with Kühner (1980) and Holland (1984), placed the subgenera of *Stropharia* and others within *Psilocybe*. Noordeloos (1995, 1999) also supports this viewpoint. The 9^th^ and 10^th^ editions of the Dictionary of the Fungi categorise *Stropharia* in *Psilocybe* (Kirk et al. 2001, 2008). In contrast, molecular studies have supported it as an independent genus (Moncalvo et al. 2002; Matheny et al. 2006; Tian and Matheny 2021).

The species of *Stropharia* have a widespread distribution all over the world. Currently, over 351 records have been listed in Index Fungorum and 185 legal names have been verified (Available online: www.indexfungorum.org, accessed on 3 July 2024). To date, only 13 species and three varieties of *Strophaira* have been reported from China: *Strophariaaeruginosa* (Curtis) Quél., *Strophariaaeruginosa* f. brunneola Hongo, *Strophariaaeruginosa* var. earthwormia T.X. Meng & Tolgor, *Strophariaalbonitens* (Fr.) Quél., *Strophariachrysocystidia* T.X. Meng & Tolgor, *Strophariahalophila* Pacioni, *Strophariahardii* G.F. Atk., *Strophariahornemannii* (Fr.) S. Lundell & Nannf., *Strophariajilinensis* T. Bau & E. J. Tian, *Stropharialignicola* E.J. Tian, *Strophariapopulicola* L. Fan, S. Guo & H. Liu, *Strophariarugosoannulata*, *Strophariarugosoannulata* f. lutea Hongo, *Strophariascabella* (Zeller) E.J. Tian & M. Gordon, *Strophariasubsquamulosa* Mitchel & A.H. Sm. and *Strophariayunnanensis* W.F. Chiu (Bau and Meng 2008; Tian and Bau 2014; Zhao 2020; Liu et al. 2021; Tian et al. 2021).

*Clitolyophyllum* is a genus within the family Lyophyllaceae; it was discovered in 2016, a Turkish species fruiting on the dead bark of *Piceaorientalis*. It is mainly characterised by fan-shaped, translucent-striate pileus; decurrent lamellae; lateral, cylindrical to flattened stipe; smooth, inamyloid spores; non-siderophilous basidia and irregular pileipellis (Sesli et al. 2016). Until now, it shows an apparently poor species diversity worldwide and currently contains only two species, of these, *Clitolyophyllumakcaabatense* Sesli, Vizzini & Contu is from Turkey and *C.umbilicatum* J.Z. Xu & Yu Li is from Gansu Province, China (Sesli et al. 2016; Xu et al. 2021).

In this study, two new species of *Stropharia* from China and a new record species of *Clitolyophyllum* from Jilin Province in China are described, based on both morphological and molecular data.

## ﻿Materials and methods

### ﻿Collection of specimens

All samples were collected during 2022–2023 from Shangping Village, Tianbaoyan National Nature Reserve, Yong’an City, Fujian Province and Red Pine King Scenic Area, Antu County, Yanbian Korean Autonomous Prefecture, Jilin Province, China. These were dried overnight by using an electric oven at 45 °C. The specimens were preserved in the Herbarium of Mycology of Jilin Agricultural Science and Technology University (**HMJU**).

### ﻿Morphological observation

Photographs of fresh basidiocarps were taken with a Canon 80D camera. The colour name and code were recorded according to Kornerup and Wanscher (1978). The micromorphology of the specimens was studied at 40×, 100×, 400×, 600× and 1000× magnifications with the help of an Olympus BX 53 (Tokyo, Japan) optical microscope (measurements were carried out at 1000× oil immersion). Sections of dried specimens were fixed in 3% potassium hydroxide (KOH), 1% Congo red and Melzer’s reagent for observation. Dimensions for basidiospores are given using the notation of the form ‘(a–)b–av–c(–d)’. The range of ‘b–c’ contains a minimum of 90% of the measured values. Extreme values, ‘a’ and ‘d’, are given in parentheses, while ‘av’ is the average value. Factor Q is the ratio of spore length to width, Qm is the average of factor Q.

### ﻿DNA extraction, PCR, sequencing and phylogenetic analyses

Total genomic DNA was extracted using an M5 Fungal Genomic DNA Kit (Mei5 Biotechnology Co., Ltd., Beijing, China) according to the manufacturers’ instructions. For polymerase chain reaction (PCR) amplification, primers ITS1 and ITS4 were used for the ITS region (White et al. 1990) and primer LR0R was paired with LR5 and LR7 to obtain the nrLSU sequences (Vilgalys and Hester 1990). The reactions were performed with the following procedure: initial denaturation at 94 °C for 5 min (ITS) or 4 min (nrLSU), 30 cycles at 94 °C for 30 s (ITS) or 40 s (nrLSU), 52 °C (nrLSU) or 53 °C (ITS) for 30 s or 45 s (nrLSU) and 72 °C for 30 s (ITS) or 40 sn(nrLSU) and, for terminal elongation, the reaction batches were incubated at 72 °C for 5 min. The PCR products were examined on 1% agarose gel, detected by JY 600 electrophoresis (Beijing JUNYI Electrophoresis Co., Ltd., Beijing, China) and then sent to BGI Co., Ltd. (Beijing, China) for sequencing.

### ﻿Phylogenetic analyses

The obtained sequences were compared with the representative ITS sequences and nrLSU sequences retrieved from GenBank. Based on previous phylogenetic studies (Moncalvo et al. 2002; Matheny et al. 2006; Liu et al. 2021; Tian and Matheny 2021; Tian et al. 2021; Zhang et al. 2021, 2024; Wang et al. 2024), other species of *Stropharia* were also included, while *Hypholomaaustrale* (Murrill) Murrill and *Hypholomafasciculare* (Huds.) P. Kumm were included as the outgroups. Sequences were aligned with MAFFT 7.0 (Katoh and Standley 2013) and edited with MEGA 7.0 (Kumar et al. 2016). The selection of the model was done by ModelFinder (Kalyaanamoorthy et al. 2017), based on the Bayesian Information Criterion (BIC). For this purpose, we chose the GTR+F+I+G4 model.

Following previous phylogenetic studies (Cai et al. 2020; Zhang et al. 2022; Qi et al. 2023), we designated *Entolomaundatum* (Gillet) M.M. Moser and *Entolomasericeum* Quél., Mém. Soc. Émul, which are closely related to this genus, as the outgroup for phylogenetic analysis. The previously described methods were used to align and edit the sequences. At the same time, for BIC, we chose the GTR+F+I+G4 model.

Maximum Likelihood (ML) analysis and Bayesian Inference (BI) analysis were used to infer the phylogenetic position of the new species. Maximum Likelihood analysis estimation was performed by IQ-TREE (Nguyen et al. 2015). BI phylogeny using Markov Chain Monte Carlo (MCMC) methods was carried out with MrBayes 3.2.2 (Ronquist et al. 2012). The significance thresholds were set to > 0.90 for Bayesian posterior probability (PP) and > 70% for ML bootstrap proportions (BP). All sequences used in this study are listed in Table 1.

**Table 1.** Specimens used in molecular phylogenetic studies and their GenBank accession numbers.

Species	Voucher	GenBank accession number		References
		ITS	nrLSU	
*Calocybecarnea*	CBS552.50	AF357028	AF223178	Hofstetter et al. (2002)
*Calocybechrysenteron*	AB10-09-142	KP192603	—	Bellanger et al. (2015)
*Calocybecoacta*	HMJU 269	OK649907	OL687156	Xu et al. (2021)
*Calocybeconvexa*	SYAU-FUNGI-007	KU528826	KU528830	Li et al. (2017)
*Calocybeconvexa*	SYAU-FUNGI-008	NR156303	NG058936	Li et al. (2017)
*Calocybegangraenosa*	Hae251.97	AF357032	AF223202	Li et al. (2017)
*Calocybeionides*	HC77/133	AF357029	AF223179	Li et al. (2017)
*Calocybenaucoria*	PAM02081103	KP192543	—	Bellanger et al. (2015)
*Calocybeobscurissima*	FR2014101	KP192650	—	Bellanger et al. (2015)
*Calocybepseudoflammula*	FR2014054	KP192579	—	Bellanger et al. (2015)
*Clitolyophyllumakcaabatense*	KATO Fungi 3184	KT934393	KT934394	Cai et al. (2020)
*Clitolyophyllumumbilicatum*	HMJU 262	OK649905	OK649873	Xu et al. (2021)
*Clitolyophyllumumbilicatum*	HMJU 1558	OK649906	OK649874	Xu et al. (2021)
***Clitolyophyllumumbilicatum***	**HMJU 5573**	**PP986998**	**PP987056**	**This study**
*Entolomaundatum*	TB7144	EF421108	AF261315	Mu et al. (2023)
*Entolomasericeum*	GLM 45918	—	AY207197	Walther et al. (2005)
*Gerhardtiacitrinolobata*	JBSD 126508	KY363576	KY363578	Vizzini et al. (2017a)
*Gerhardtiahighlandensis*	PBM2806 (CUW)	GU734744	EF535275	Cai et al. (2020)
*Hypholomaaustrale*	PBM3481	HQ832446	HQ832456	Tian et al. (2021)
*Hypholomafasciculare*	TJB10226	HQ222023	HQ832457	Tian et al. (2021)
*Hypsizygusulmarius*	DUKE-JM/HW	EF421105	AF042584	Cai et al. (2020)
*Lyophyllummaleolens*	AB11-11-328	KP192607	—	Bellanger et al. (2015)
*Lyophyllumsykosporum*	IFO30978	AF357050	AF223208	Hofstetter et al. (2002)
*Lyophyllumtransforme*	GC08101108	KP192653	—	Bellanger et al. (2015)
*Myochromellaboudieri*	BSI96/84	AF357047	DQ825430	Cai et al. (2020)
*Myochromellainolens*	CBS330.85	AF357045	AF223201	Hofstetter et al. (2002)
*Ossicaulisborealis*	SYAU-FUNGI-076	OP782047	OP782284	Qi et al. (2023)
*Ossicaulisborealis*	SYAU-FUNGI-079	OP782050	OP782285	Qi et al. (2023)
*Ossicaulislignatilis*	D604	DQ825426	AF261397	Cai et al. (2020)
*Ossicaulisyunnanensis*	IJ152	KY411962	KY411960	Yang et al. (2017)
*Ossicaulisyunnanensis*	IH26	KY411961	KY411959	Yang et al. (2017)
*Sagaranellagibberosa*	CBS328.50	AF357041	AF223197	Hofstetter et al. (2002)
*Sagaranellatylicolor*	BSI92/245	AF357040	AF223195	Hofstetter et al. (2002)
*Strophariaacanthostipitata*	JLCLD4-120329-01	MF882993	MF882995	Vizzini et al. (2017b)
*Strophariaacanthostipitata*	JBSD127401	NR156637	NG060022	Vizzini et al. (2017b)
*Strophariaaeruginosa*	HMJAU 4789	MW492533	MW492636	Tian et al. (2021)
*Strophariaaeruginosa*	HMJAU 22865	MW492534	MW492637	Tian et al. (2021)
*Strophariaalbonitens*	FO46892	—	AF291368	Wei et al. (2001)
*Strophariaalbonitens*	G0187	—	MK278582	Varga et al. (2019)
*Strophariaambigua*	PBM 2257	AY818350	AY646102	Yang et al. (2005)
*Strophariaatroferruginea*	HU32915	MK141060	MK434168	Khan et al. (2019)
*Strophariaatroferruginea*	HU32916	MK141061	MK433557	Khan et al. (2019)
*Strophariacaerulea*	BJTC FM225	MZ577604	—	Liu et al. (2021)
*Strophariacaerulea*	BJTC FM1177	MZ577597	—	Liu et al. (2021)
*Strophariacaerulea*	BJTC FM1449	MZ577571	—	Liu et al. (2021)
*Strophariacaerulea*	BJTC FM1512	MZ577579	—	Liu et al. (2021)
*Strophariacoronilla*	CBS 534.50	MH856747	MH868269	Vu et al. (2019)
*Strophariahardii*	TENN-F-071760	MW821365	MW821382	Tian et al. (2021)
*Strophariahardii*	SV S3	—	AF261636	Moncalvo et al (2002)
*Strophariahardii*	SV S7	—	AF261637	Moncalvo et al (2002)
*Strophariahornemannii*	TRTC156845	JN021094	—	Dentinger et al. (2011)
*Strophariahornemannii*	TRTC150931	JN021093	—	Dentinger et al. (2011)
*Strophariahornemannii*	TRTC150919	JN021092	—	Dentinger et al. (2011)
*Strophariainuncta*	GLM46029	—	AY207303	Walther et al. (2005)
*Strophariainuncta*	NL-5406	—	MK278584	Varga et al. (2019)
*Strophariajilinensis*	HMJAU 22486	JF961347	—	Tian and Bau (2014)
*Stropharialignicola*	T17(HMJAU 37429)	MW492530	MW492633	Tian et al. (2021)
*Stropharialignicola*	Ti4(HMJAU 37510)	MW492531	MW492634	Tian et al. (2021)
*Strophariamammillata*	CBS 535.50	MH856748	MH868270	Vu et al. (2019)
*Strophariamelanosperma*	OMDL K	OR945032	—	Unpublished
*Strophariamelanosperma*	S.D. Russell iNaturalist # 91080138	OM972388	—	Unpublished
***Strophariamicroaeruginosa***	**HMJU 12422**	**PP702369**	**PP702382**	This study
***Strophariamicroaeruginosa***	**HMJU 12635**	**PP715434**	**PP715435**	This study
*Strophariapopulicola*	BJTC FM1483	MZ661117	MZ661121	Liu et al. (2021)
*Strophariapopulicola*	BJTC FM1480	MZ661094	MZ661118	Liu et al. (2021)
*Strophariapopulicola*	HSA361	MZ661093	MZ661119	Liu et al. (2021)
*Strophariarugosoannulata*	Z3(HMJAU46972)	MW492535	MW492640	Tian et al. (2021)
*Strophariarugosoannulata*	HMJAU25602	MW492537	MW492639	Tian et al. (2021)
*Strophariarugosoannulata*	ACD0479	—	OP235390	Unpublished
***Strophariasubrugosoannulata***	**HMJU 12439**	**PP702370**	**PP702383**	This study
***Strophariasubrugosoannulata***	**HMJU 12441**	**PP702371**	**PP702384**	This study
*Tephrocybeconfusa*	GC08110114	KP192633	—	Bellanger et al. (2015)
*Tephrocybellaconstrictospora*	TO HG3329	MF614962	MF614963	Hyde et al. (2017)
*Tephrocybellagriseonigrescens*	TO HG21112014	NR137975	KR476785	Crous et al. (2015)

Note: Newly generated sequences are in bold.

## ﻿Results

### ﻿Phylogenetic analyses

**Figure 1.**
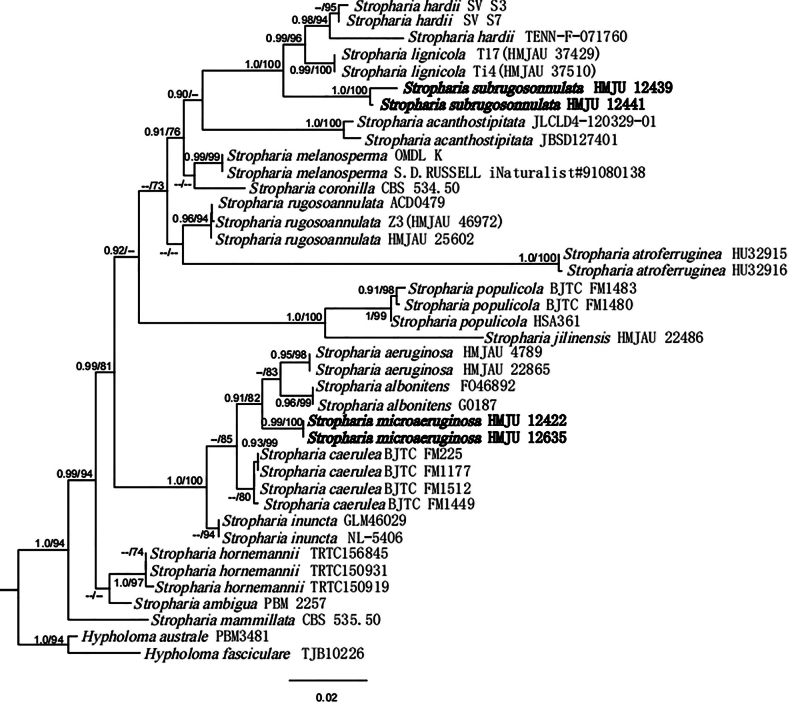
ML and Bayesian phylogenetic analysis of *Stropharia*, based on ITS and nrLSU sequences. This study species is in bold.

**Figure 2.**
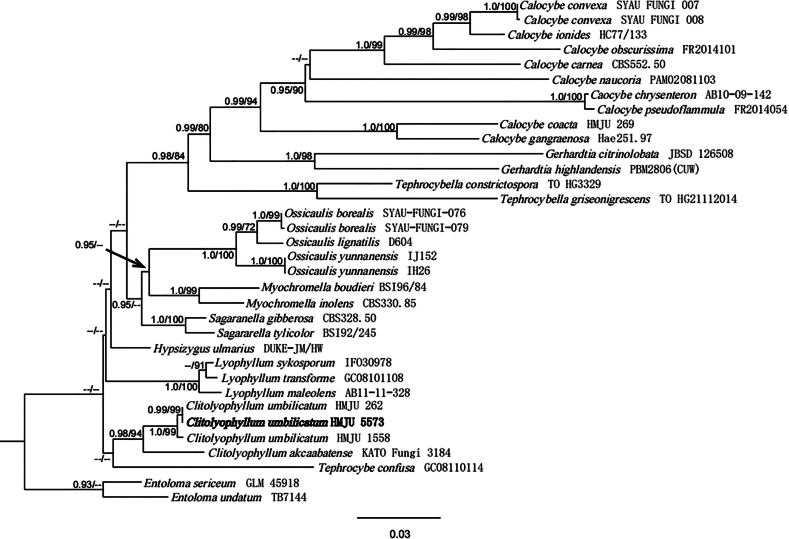
ML and Bayesian phylogenetic analysis of *Clitolyophyllum* based on ITS and nrLSU sequences. This study species is in bold.

The combined dataset included 123 sequences, of which 113 were retrieved from GenBank. Both ML and BI methods produced the same tree topology, thus, only the ML tree is shown in Figs 1, 2. In both figures, Bayesian PP values (left) and MLBP values (right) are presented near each node. In the BLAST results, the *S.subrugosoannulata* sequences showed 92.84% similarity to *S.hardii* (OP679883) with 89% query coverage for ITS and 99.41% similarity to *S.lignicola* (NG079687), with 100% query coverage for nrLSU. The *S.microaeruginosa* sequences showed 96.88% similarity to *S.aeruginosa* (OR336166) with 97% query coverage for ITS and 99.69% similarity to *S.aeruginosa* (MK278581) with 94% query coverage for nrLSU; The *C.umbilicatum* sequences showed 99.38% similarity to *C.umbilicatum* (OK649905) for ITS.

Phylogenetic analyses indicate that the specimens from south-eastern China are in two separate clades with a high degree of support, which suggests that they represent two distinct new species. *S.subrugosoannulata* clusters with *S.hardii* and *S.lignicola*, implying they were phylogenetically closely related to each other. *S.microaeruginosa*, *S.aeruginosa* and *S.albonitens* appear to be the most closely related species (Fig. 1). According to the results of the phylogenetic analysis, the voucher HMJU 5573 was clustered with *C.umbilicatum* (BPP = 0.99, MLBP = 99) (Fig. 2).

### ﻿Taxonomy

#### ﻿ Stropharia subrugosoannulata

Taxon classificationFungiAgaricalesStrophariaceae

J.Z. Xu sp. nov.

4710F19A-9152-5249-AC50-C2A87686D748

Fungal Names: FN 572029

Fig. 3

##### Diagnosis.

*Strophariasubrugosoannulata* is distinguished from other species of the genus *Stropharia* by the pileus covered with greyish-orange squamules in the centre, stipe light brown and surface covered with white triangular squamules. Acanthocytes present in the basal mycelium of stipe. Chrysocystidia rare.

**Figure 3.**
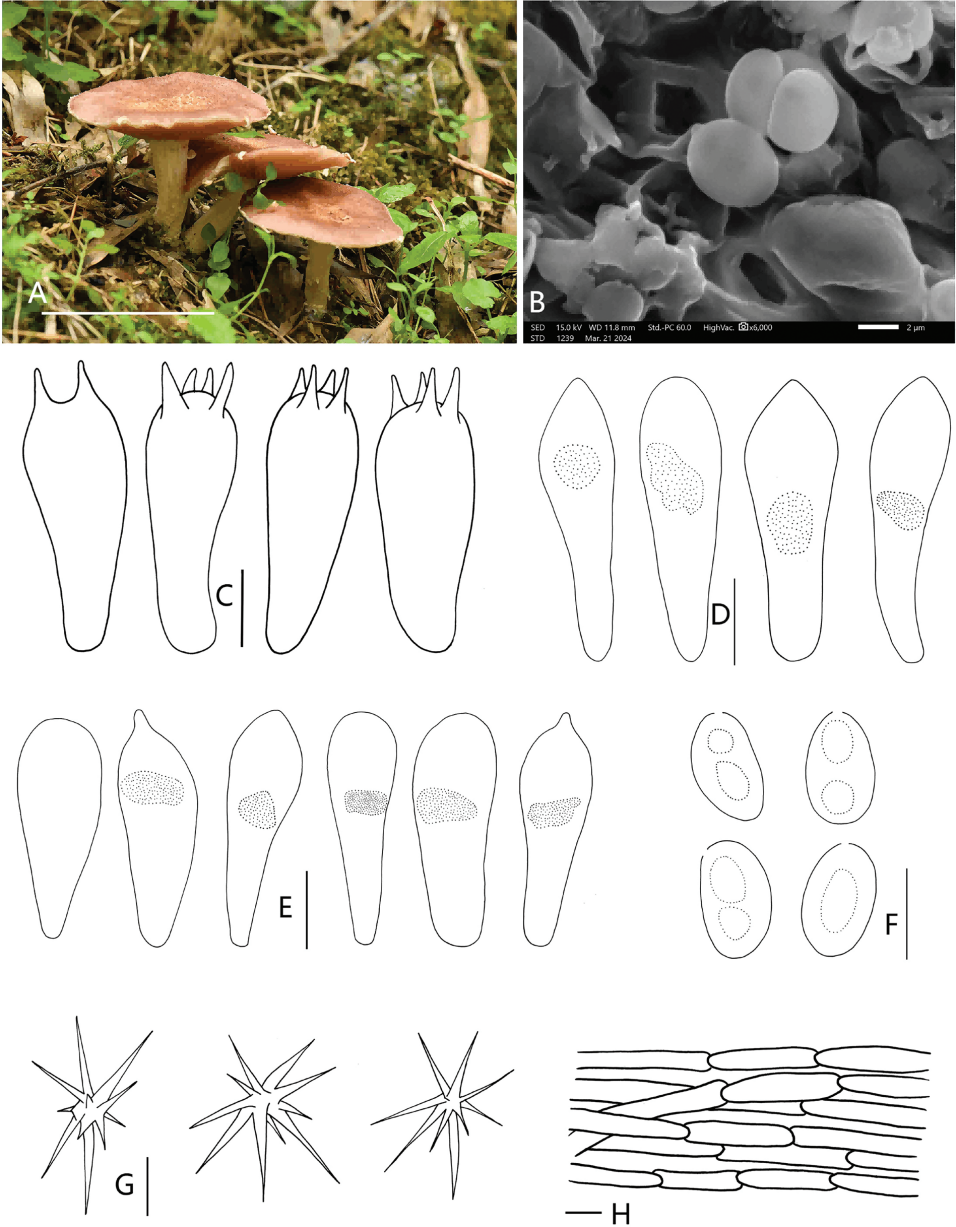
*Strophariasubrugosoannulata* (HMJU 12441, holotype) **A** habitat and basidiocarps **B** SEM images of basidiospores **C** basidia **D** cheilocystidia **E** pleurocystidia **F** basidiospores **G** acanthocytes **H** pileipellis. Scale bars: 5 cm (**A**); 5 µm (**C, F**); 10 µm (**D, E, G, H**).

##### Holotype.

China, Fujian Province, Sanming City, Tianbaoyan Nature Reserve, Longwu Village, on soil, Phyllostachys pubescens, alt. 700 m, 21 October 2023, J.P. Liao (HMJU 12441, holotype).

##### Etymology.

“sub” means “near”, named as it is similar to *S.rugosoannulata*.

##### Description.

Pileus 25–60 mm diam., planoconcave or almost plane with or without being depressed in the centre, red copper to dull red (7C3–8B3), covered with greyish-orange (7B5) squamules in the centre, the margins with partial veil remnants. Lamellae adnate to adnexed, crowded, titian red to terra-cotta (7D6–7E7), with lamellulae in 1–3 tiers. Stipe 25–48 mm long and 6–10 mm wide, sometimes evanescent annulus in the upper part of the stipe, slightly broad with whitish rhizoids at the base. Surface longitudinally striate, light brown (6D6) and covered with white triangular squamules.

Basidiospores [30/4/3] (5.3) 5.8–6.3–6.8 (7.0) × (3.2) 3.4–3.7–4.0 (4.1) µm, Q = (1.50) 1.56–1.86 (1.91), Qm = 1.72, ellipsoid to subovate, obvious germ-pore, fawn to light fawn in KOH and Melzer’s reagent. Contains 1–2 guttulates. Basidia (10.6) 13.8–16.8–19.4 (19.9) × (4.1) 5.6–6.8–7.7 (8.7) µm. 2–4 spored, clavate, sterigmata up to 2.6 µm long, hyaline in KOH. Pleurocystidia (24.0) 26.3–30.5–36.9 (39.0) × (7.9) 8.3–10.1–12.8 (13.9) µm, clavate with or without umbo, hyaline in KOH, containing amorphous contents. Cheilocystidia (27.9) 29.1–33.2–37.1 (41.4) × (8.8) 9.0–10.7–13.0 (15.5) μm. Clavate, expanding at the tip, tapering downwards, sometimes curved. Chrysocystidia is rare. Lamellae trama regular, parallel to subparallel, 5.1–12.7 μm wide, hyaline in KOH. Pileipellis a cutis of parallel, hyphae 5.1–11.6 µm wide. Acanthocytes present in the basal mycelium of stipe. Clamp connections are present.

##### Habitat.

Gregarious on the soil in the phyllostachys pubescens.

##### Known distribution.

Known only from south-eastern China.

##### Additional material examined.

Fujian Province, Sanming City, Tianbaoyan Nature Reserve, Longwu Village, on soil, Phyllostachys pubescens alt. 700 m, 21 October 2023, J.P. Liao (HMJU 12439).

##### Comments.

The species is characterised mainly by the pileus covered with greyish-orange squamules in the centre, the margins with partial veil remnants, stipe light brown and surface covered with white triangular squamules, Sometimes evanescent annulus in the upper part of the stipe, chrysocystidia rare.

#### ﻿ Stropharia microaeruginosa

Taxon classificationFungiAgaricalesStrophariaceae

J.Z. Xu sp. nov.

AEF26735-4898-5764-B42F-3A78323C724B

Fungal Names: FN 572030

Fig. 4

##### Diagnosis.

*Strophariamicroaeruginosa* pileus bluish-grey when young becoming lighter toward margins, later greyish-turquoise lightening towards the edges and surface radially striate when young, lamellae adnate to subdecurrent, stipe with white squamules at the base, acanthocytes absent making it unique amongst the *Stropharia* species.

**Figure 4.**
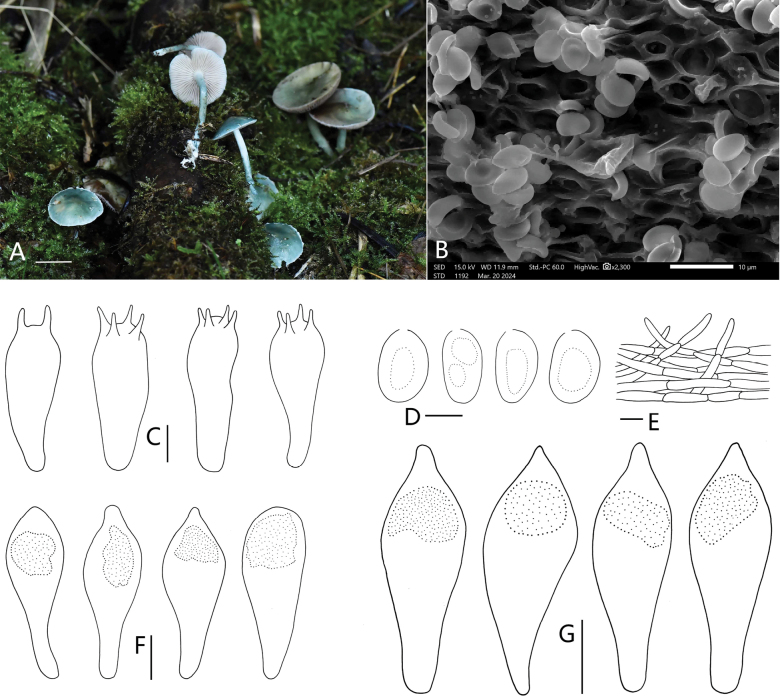
*Strophariamicroaeruginosa* (HMJU 12635, holotype). **A** habitat and basidiocarps **B** SEM images of basidiospores **C** basidia **D** basidiospores **E** pileipellis **F** pleurocystidia **G** cheilocystidia. Scale bars: 2 cm (**A**); 5 µm (**C, D**); 10 µm (**E, F, G**).

##### Holotype.

China, Fujian Province, Sanming City, Tianbaoyan Nature Reserve,Shangping Village, scattered in the moss under mixed forests dominated by phyllostachys pubescens and coniferous forest, alt. 1100 m, 31 October 2023, J.P. Liao (HMJU 12635, holotype).

##### Etymology.

“micro” means “small”, referring to the small basidiocarps and the margins often with partial veil remnants.

##### Description.

Pileus 27–38 mm diam., centre of pileus bluish-grey (20F3) when young becoming lighter towards margins, later greyish-turquoise (24D4) lightens toward the edges, initial convex, the edge of the pileus upturned at maturity, smooth surface, viscid to glutinous, the margins often with partial veil remnants, submembranous, surface radially striate when young, gradually becomes less obvious as it matures. Lamellae adnate to subdecurrent, moderately crowded, grey (15C1), with lamellulae in 1–3 tiers. Stipe 51–75 mm long and 3.7–6.0 mm wide, concolorous with the pileus and lightening upwards, white squamules at the base, sometimes evanescent annulus in the upper part of the stipe. Further, slightly broad and with whitish rhizoids at the base.

Basidiospores [30/4/3] (5.4) 6.2–7.3–8.2 (8.8) × (3.7) 4.0–4.3–4.9 (5.0) µm, Q = (1.10) 1.42–1.99 (2.06), Qm = 1.69, elliptical, with an obvious germ-pore, light brown in KOH. Contains 1–2 guttulates. Basidia (16.4) 17.9–21.0–23.9 (27.0) × (5.2) 5.9–7.3–9.0 (9.9) µm, 2–4 spored, clavate, sterigmata up to 3.4 µm long, hyaline in KOH. Pleurocystidia (27.6) 33.5–39.9–46.3 (47.4) × (10.2) 11.1–13.6–16.0 (16.3) μm, clavate, with or without short mucronate apex, with an amorphous highly refractive content distributed in enlarged or raised areas. Cheilocystidia (29.8) 30.7–36.1–41.4 (45.6) × (7.9) 8.4–11.6–14.4 (16.1) μm. clavate, with homogenous content, mucronate at the apex, expanded apically, tapering downwards, sometimes curved. Chrysocystidia rare. Lamellae trama regular, subparallel, 6.7–14.3 µm wide, hyaline in KOH. Pileipellis a cutis of subparallel, slightly upturned, hyphae 2.7–5.5 µm wide. Clamp connections are present.

##### Habitat.

Scattered in the moss under mixed forests dominated by phyllostachys pubescens and coniferous forest

##### Known distribution.

Known only from south-eastern China.

##### Additional material examined.

Fujian Province, Sanming City, Tianbaoyan Nature Reserve, Shangping Village, scattered in the moss under mixed forests dominated by phyllostachys pubescens and coniferous forest, alt. 1100 m, 31 October 2023, J.P. Liao (HMJU 12422).

##### Comments.

This species is characterised mainly by the centre of pileus bluish-grey when young becoming lighter toward margins, later greyish-turquoise lightening towards the edges, the margins often with partial veil remnants, surface radially striate, stipe concolorous with the pileus and white squamules at the base, the spores with an obvious germ pore and chrysocystidia rare.

#### ﻿ Clitolyophyllum umbilicatum

Taxon classificationFungiAgaricalesLyophyllaceae

J.Z. Xu & Yu Li, Journal of Fungi 7 (12, no. 1101): 9 (2021)

BA557639-84B6-520E-AA5D-40282C857CEC

Fig. 5

##### Description.

Basidiocarps omphalioid or clitocyboid. Pileus 30–50 mm in diam., deeply depressed, pale orange to greyish-brown (6A3-6D3), margin incurved with white appendages, slightly wavy with age. Lamellae decurrent, moderately crowded, thin, bluish-grey to grey (20D2-19E1), with numerous tiers of lamellulae, edges entire. Stipe 40–60 mm long and 5–8 mm wide, central, cylindrical or slightly compressed, equal or slightly tapering towards the apex, surface brownish-grey to dark brown (6E2-6F5), radially striate. Context thin, fleshy.

**Figure 5.**
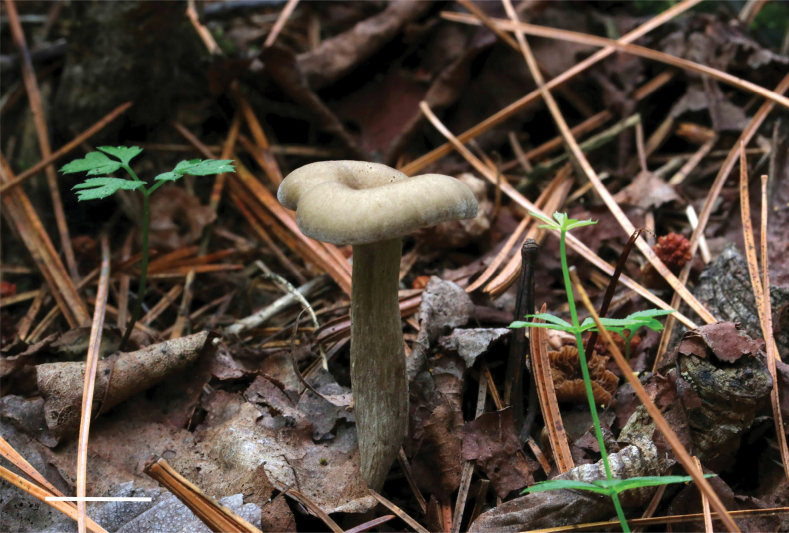
Basidiomes of *Clitolyophyllumumbilicatum* (HMJU 5573). Scale bars: 2 cm.

Basidiospores [30/4/3] (4.5) 4.6–6.3–8.2 (8.6) x (3.1) 3.4–4.3–5.3 (5.8) μm, Q = (1.17) 1.18–1.89 (2.10), Qm = 1.50, subglobose-ellipsoid, smooth, inamyloid, cyanophilic. Basidia (20.6) 22.6–25.6–28.4 (28.6) x 5.4–7.0–8.7 (8.8) μm, narrowly clavate or clavate, 2–4 spores, with siderophilous granulations. Hymenophoral trama regular, hyphae 2.5–16.7 μm wide. Hymenial cystidia not observed. Pileipellis a cutis of subparallel, dense, cylindrical hyphae, hyphae 2.6–16.5 μm wide, thin-walled, irregular. Stipitipellis made up of regularly parallel, hyphae 3.3–17.5 μm wide. Clamp connections present.

##### Habitat.

Scattered on soil under mixed forests

##### Known distribution.

The species is known to be distributed in China.

##### Specimens examined.

China, Jilin Province, Yanbian Korean Autonomous Prefecture, Antu County, Red Pine King Scenic Spot, 31 July 2022, J.Z. Xu HMJU 5573.

##### Comments.

This species was originally described from Gansu Province and is characterised by the omphalioid or clitocyboid habit, umbilicate pileus, central stipe, smooth, inamyloid spores and subregular pileipellis (Xu et al. 2021).

## ﻿Discussion

Morphologically, *S.rugosoannulata* is the most similar species to *S.subrugosoannulata* in pileus margins with partial veil remnants, lamellae adnate, stipe equal or slightly tapered upwards and with annulus, whitish rhizoids at the base. The difference between *S.subrugosoannulata* and *S.rugosoannulata* is that the *S.rugosoannulata* has a larger pileus (50–150 mm), smooth, lamellae are white when young, turning dark brown or almost black with age and larger basidiospores (Murrill 1922). *S.scabella* showed similarities with *S.subrugosoannulata* in lamellae adnate and light yellow pileus, but *S.scabella* hemispherical to convex pileus, annulus obvious, inconspicuous germ-pore (Tian and Matheny 2021). *S.jilinensis* bears resemblance to *S.subrugosoannulata* due to the pileus covered with yellowish-brown squamules, the margins with partial veil remnants, but *S.jilinensis* has grey violet to yellowish-brown pileus, white stipe (Tian and Bau 2014). Phylogenetically, *S.subrugosoannulata* is closely related to *S.hardii* (Atkinson 1906) and *S.lignicola* (Tian et al. 2021) (Fig. 1). *S.hardii* differs from *S.subrugosoannulata* in that it does not have acanthocytes, spores purple-brown, the spores without an obvious germ pore (Atkinson 1906). *S.lignicola* also can be easily distinguished from *S.subrugosoannulata* by the pileus grey-yellow, incurved margin and stipe surface covered with recurved yellowish squamules towards the base (Tian et al. 2021).

*S.microaeruginosa* is very similar to *S.aeruginosa* in morphology; however, when compared that to the new species, *S.aeruginosa* basidiomata medium to large, annulus evident, acanthocytes present in the basal mycelium of stipe, the spores without an obvious germ pore (Zhao 2020). *S.populicola* bears resemblance to *S.microaeruginosa* due to pileus margins with partial veil remnants, lamellae adnate, sometimes evanescent annulus of the stipe, but *S.populicola* pileus non-sticky, pleurocystidia rare, acanthocytes present on the basal mycelium at stipe (Liu et al. 2021). *S.microaeruginosa* is similar to *Strophariavenusta* P.S. Silva, Cortez & R.M. Silveira in pileus viscid and the margins often with partial veil remnants, stipe with squamules, gill trama regular, but *S.venusta* pileus reddish-brown, lamellae adnexed to sinuate, acanthocytes present abundantly on rhizomorphs’ surface, cheilochrysocystidia absent (Da Silva et al. 2009). Phylogenetic analysis indicate that *S.microaeruginosa* is sister to *S.aeruginosa* and *S.albonitens* (Fig. 1); *S.albonitens* also can be distinguished from *S.microaeruginosa* by the basidiospores purple-brown, the spores without an obvious germ pore (Karsten 1879).

The specimen from Jilin Province shares the following characteristics with *C.umbilicatum* (Xu et al. 2021) in the original description: Basidiocarps omphalioid or clitocyboid. Pileus deeply depressed, margin slightly wavy with age. Lamellae decurrent, moderately crowded, thin, with numerous tiers of lamellulae, edges entire. Basidiospores subglobose-ellipsoid, smooth, inamyloid, cyanophilic. Basidia, narrowly clavate or clavate, with siderophilous granulations. Hymenial cystidia not observed. Clamp connections present. However, in the original description of *C.umbilicatum*, pileus surface smooth with radially striate, slightly hygrophanous. Phylogenetic analyses show that *C.umbilicatum* and *C.akcaabatense* are closely related (Fig. 2), but *C.akcaabatense* differs in that the lamellae at first whitish then light cream or beige, Stipe eccentric, Caulocystidia present (Sesli et al. 2016). Therefore, combining morphological and microscopic features, the specimen from Jilin was *C.umbilicatum*.

Until now, a total of 15 species and three varieties of *Strophaira* have been reported from China. On the basis of observations and literature (Bau and Meng 2008; Meng 2008; Tian and Bau 2014; Zhao 2020; Liu et al. 2021; Tian et al. 2021), a key for the *Strophaira* species from China is provided.

### ﻿Key to the species of *Stropharia* known from China

**Table:** 

1	Pileus dry or slightly viscid when wet	**2**
–	Pileus viscid to glutinous	**6**
2	Basidiospores subhexagonal in side view	**3**
–	Basidiospores non-subhexagonal in side view	**5**
3	Pileus dark red to reddish-brown	***S.rugosoannulata***
–	Pileus yellowish-brown to pale yellow to yellowish-white	**4**
4	Cheilocystidia or pleurocystidia as chrysocystidia, on enriched soil	**13**
–	Cheilocystidia as leptocystidia, rarely chrysocystidia, on saline-alkali or barren soil	***S.halophila***
5	basidiospores with a conspicuous germ pore	**14**
–	basidiospores with an inconspicuous germ pore	**17**
6	Hymenial acanthocytes present	***S.lignicola***
–	Hymenial acanthocytes absent	**7**
7	Pileus without green tone	**8**
–	Pileus with green tone	**10**
8	Pileus brown or pale brown	**9**
–	Pileus dark brown or orange-yellow	**13**
9	Basidiospores without a germ pore, black brown in KOH	***S.subsquamulosa***
–	Basidiospores with a germ pore, slightly dark brown in KOH	***S.albonitens***
10	Cheilocystidia as leptocystidia	**11**
–	Cheilocystidia as chrysocystidia	***S.chrysocystidia***
11	Cheilocystidia clavate with a dull to capitate apex	**12**
–	Cheilocystidia flexuously cylindrical with a branched apex	***S.aeruginosa var. earthwormia***
12	Pileus fading brown or clay colour in age	***S.aeruginosa f. brunneola***
–	Pileus greyish-green with yellowish margin in age	**15**
13	Lamellae greyish-purple or purple grey brown	**18**
–	Lamellae pale grey to pale cinnamon	***S.rugosoannulata f.lutea***
14	Basidiospores black purple brown	***S.yunnanensis***
–	Basidiospores fawn to dark yellow	**16**
15	Cystidia as chrysocystidia	***S.aeruginosa***
–	Chrysocystidia rare	***S.microaeruginosa***
16	Lamellae titian red to terra-cotta	***S.subrugosoannulata***
–	Lamellae purple grey to pale cinnamon	***S.hornemannii***
17	Cheilocystidia as chrysocystidia and pleurocystidia from one show larger hollow pattern	***S.scabella***
–	Cheilocystidia with branch or spherical chain of cells arranged	***S.jilinensis***
18	Pleurocystidia rare	***S.populicola***
–	Pleurocystidia as chrysocystidia	***S.hardii***

## Supplementary Material

XML Treatment for Stropharia subrugosoannulata

XML Treatment for Stropharia microaeruginosa

XML Treatment for Clitolyophyllum umbilicatum

## ﻿Additional information

### Author ORCIDs

Shi-Chen Huang https://orcid.org/0000-0001-5736-142X

Ji-Ze Xu https://orcid.org/0000-0003-2082-8529

### Data availability

All of the data that support the findings of this study are available in the main text.
